# Association between HLA-DQ Gene Polymorphisms and HBV-Related Hepatocellular Carcinoma

**DOI:** 10.1155/2017/7150386

**Published:** 2017-07-06

**Authors:** Jingzhu Lv, Tao Xu, Zhongqing Qian, Hongtao Wang

**Affiliations:** ^1^Department of Biochemistry and Molecular Biology, Bengbu Medical College, Bengbu 233003, China; ^2^Department of Clinical Laboratory, Bengbu Medical College, Bengbu, Anhui 233030, China; ^3^Department of Microbiology and Immunology, Medical School of Southeast University, Nanjing, Jiangsu 210009, China; ^4^Clinical Testing and Diagnose Experimental Center of Bengbu Medical College, Bengbu, Anhui 233030, China; ^5^Department of Immunology, Bengbu Medical College, Bengbu Anhui 233030, China; ^6^Anhui Key Laboratory of Infection and Immunity, Bengbu Medical College, Bengbu Anhui 233030, China

## Abstract

Hepatocellular carcinoma (HCC) is one of the most common causes of cancer-related mortality worldwide. Host gene variants may influence hepatitis B virus- (HBV-) related HCC. Human leukocyte antigens (HLA) play an important role in presenting virus antigens to immune cells that are responsible for the clearance of virus-infected cells and tumor cells. Previous studies have investigated the HLA-DQ (rs2856718 and rs9275572) polymorphisms that may be associated with the development of HBV-related HCC. However, the results are controversial or inconclusive. Hence, we conducted a meta-analysis to derive a more precise estimation of the associations. A total of 6 articles were used to evaluate the effect of the two polymorphisms on the risk of HBV-related HCC. Odds ratios (ORs) and 95% confidence intervals (95% CIs) were calculated. We found that rs2856718 and rs9275572 in HLA-DQ significantly decreased HBV-related HCC in total population, especially in Chinese, but not in Saudi Arabian. Further validation of our results in larger populations and different ethnicities are required.

## 1. Introduction

Primary liver cancer is one of the six most common cancers and the second largest cause of cancer deaths worldwide (746,000 cases or 9.1% of all cancer deaths), especially in undeveloped countries [1, 2]. Approximately 75% of liver cancers occur in Asia, whereas China alone accounts for more than 50% of all cases [3]. Globally, the vast majority of histologic types of primary liver cancers (approximately 80%) are hepatocellular carcinoma (HCC), a malignant tumor arising from hepatocytes, the liver's parenchymal cells [4]. It is estimated that 75%–85% of HCC patients are attributable to chronic infections with hepatitis B virus (HBV), especially in Asian populations and particularly in Chinese [5]. Besides HBV infection, other extrinsic factors, such as alcohol, smoking, physical inactivity, chemical exposure, and poor dietary habits, are also involved in developing HCC [6]. However, only a small fraction of infected patients can progress to HCC during their lifetime. So intrinsic factors, such as genetic mutations, may be vital for tumor development [7, 8]. Furthermore, genetic epidemiology points out that genetic polymorphisms of genes involving in different processes of carcinogenesis may also play an important role to determine individual susceptibility to HCC and improve the prevention and treatment of this cancer [9–11].

Human leukocyte antigen (HLA) has been identified to be associated with regulating the immune response to HBV infection and clinical outcomes [12]. HLA-DQs are highly polymorphic especially in exon 2, which codes for antigen-binding sites. The single-nucleotide polymorphism (SNP) rs2856718 locates in the intergenic region between HLA-DQA2 and HLA-DQB1. Hu et al.'s study showed that HLA-DQ rs2856718 significantly decreased the host HCC risk [13]. The SNP rs9275572 locates between HLA-DQA and HLA-DQB on 6p21.32. A recent genome-wide association study (GWAS) indicated that the HLA-DQ rs9275572 polymorphism was significantly associated with HCV-related HCC in Japanese population [14]. Chen et al.'s study found that HLA-DQ rs9275572 polymorphism may have a protective impact on HBV-related HCC [15].

Previous studies have assessed the association between HLA-DQ (rs2856718 and rs9275572) polymorphisms and HBV-related HCC susceptibility, but the results of previous studies are inconsistent and inconclusive [13, 15–19]. Therefore, we performed a comprehensive meta-analysis to derive a more precise estimation of the relationship between HLA-DQ (rs2856718 and rs9275572) polymorphisms and HBV-related HCC risk. To the best of our knowledge, this is the first meta-analysis to analyze the association of the HLA-DQ (rs2856718 and rs9275572) polymorphisms with HBV-related HCC risk.

## 2. Material and Methods

### 2.1. Search Strategy

To identify relevant studies, we systematically searched PubMed, EMBASE, Google Scholar and China National Knowledge Infrastructure (CNKI) databases. The search strategy was based on a combination of “HLA-DQ,” “hepatitis B virus,” or “HBV”; “Hepatocellular carcinoma,” “HCC,” or “liver cancer”; “polymorphism” or “SNP”; and “rs2856718” or “rs9275572” (up to March 27, 2017). The languages of the reviewed articles were limited to English and Chinese. In addition, references of retrieved articles were also screened.

### 2.2. Inclusion and Exclusion Criteria

The following criteria were necessary for inclusion in the meta-analysis: (1) a case-control study that had investigated the genetic risk of HBV-related HCC in relation to HLA-DQ rs2856718 or rs9275572, (2) original papers containing independent data, (3) the study that provided the available genotype frequencies, (4) the study that provided sufficient information for calculating the pooled odds ratios (ORs) with 95% confidence intervals (CIs), and (5) the genotype distribution of a control group that are consistent with the Hardy-Weinberg equilibrium (HWE).

Exclusion criteria were as follows: (1) case-only studies, (2) review articles, (3) repetitive reports, and (4) lack of genotype frequency data. In addition, if multiple studies had overlapping data, only the most recent version was used.

### 2.3. Data Extraction

The following data were independently extracted from each study by two authors (Jingzhu Lv and Tao Xu): the first author's name, year of publication, country, genotyping method, number of cases and controls, genotype, and allele frequency. After extraction, data were reviewed and compared by the two independent investigators. Any disagreements were resolved by discussion with the third investigator.

### 2.4. Statistical Analysis

All statistical analyses were performed using Stata software version 12.0 (Stata Corporation, College Station, TX). The Hardy-Weinberg equilibrium test in the control group was undertaken using the *χ*^2^-test (*P* < 0.05 was considered as significant disequilibrium in the control group). Odds ratios (ORs) with 95% confidence intervals (95% CIs) were used to estimate the strength of the association between HLA-DQ (rs2856718 and rs9275572) polymorphisms and HBV-related HCC. The pooled ORs were obtained from combination of single studies by homozygote comparison, heterozygote comparison, dominant and recessive models, and allele comparison, respectively. The significance of pooled ORs was assessed by the Z test, and *P*_Z_ < 0.05 was considered as the significance threshold. Based on the heterogeneity test, the pooled OR was calculated using the fixed (*P*_H_ ≥ 0.05, *I*^2^ ≤ 50%) or random (*P*_H_ < 0.05, *I*^2^ > 50%) effect models. Sensitivity analysis was carried out to identify the effect of data from each study on the pooled ORs. Finally, publication bias was assessed using Egger's test, and *P*_E_ < 0.05 was considered statistically significant.

## 3. Results

### 3.1. Characteristics of the Included Studies

**Figure 1:**
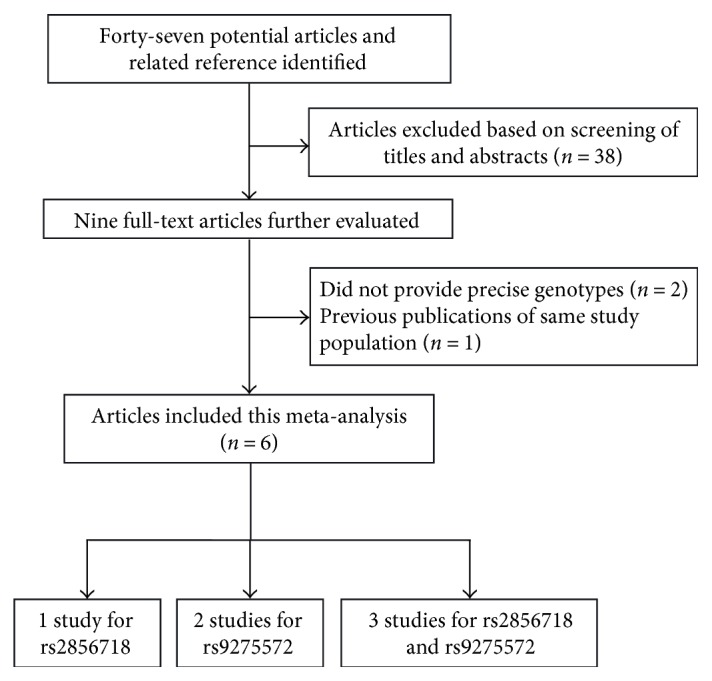
The flow charts of literature search and study selection.

A total of 47 potentially relevant articles published up to March 27, 2017 were systematically identified in PubMed, EMBASE, Google Scholar, and CNKI. The flow chart that summarized the literature review process and the specific reasons for any exclusion from the meta-analysis are shown in Figure 1(). In the end, 6 articles that met the inclusion criteria were included in the meta-analysis of 1 study for HLA-DQ rs2856718, 2 studies for HLA-DQ rs9275572, and 3 studies for HLA-DQ rs2856718 and HLA-DQ rs9275572 [13, 15–19]. Characteristics of all eligible articles are shown in Table 1. Of the 6 articles, 4 articles including 1820 cases, 2266 CHB, and 2601 controls evaluated the association between HLA-DQ rs2856718 and HBV-related HCC risk, while 5 studies evaluated the association between the HLA-DQ rs9275572 and HBV-related HCC risk (1092 cases, 1920 CHB, and 1646 controls).

### 3.2. Meta-Analysis of the Association between HLA-DQ rs2856718 and HBV-Related HCC Risk

**Figure 2:**
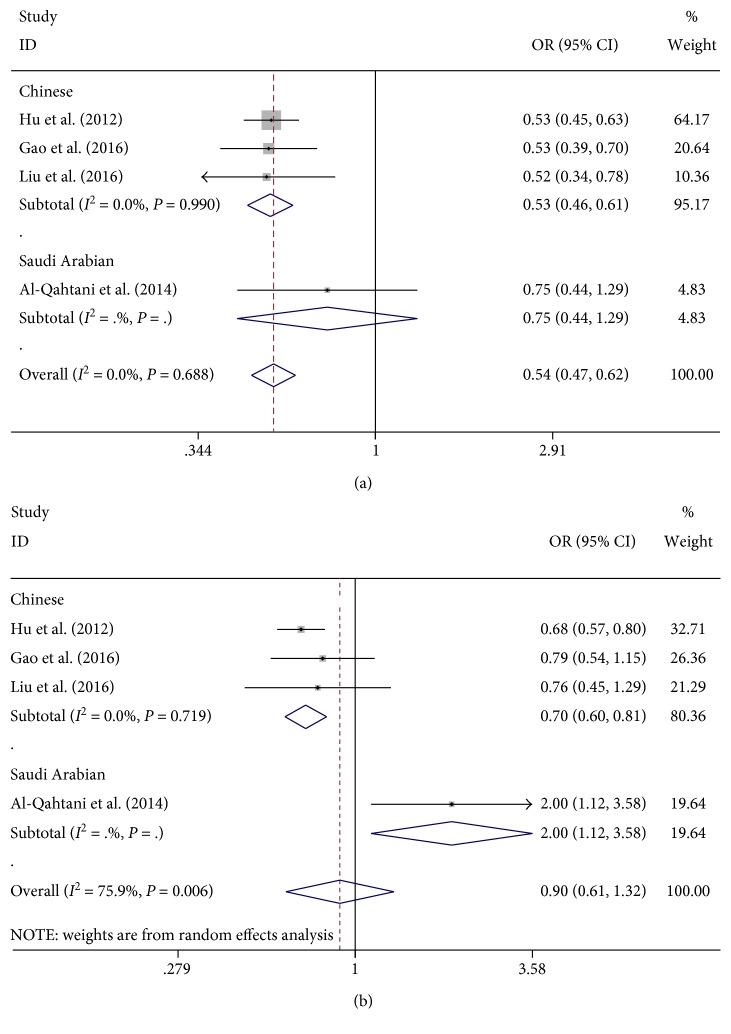
Forest plots for HLA-DQ rs2856718 polymorphism and the risk of HBV-related HCC. (a) Overall meta-analysis of the relationship between HLA-DQ rs2856718 polymorphism and HBV-related HCC (HCC versus control) risk in dominant model (AG + GG versus AA). (b) Overall meta-analysis of the relationship between HLA-DQ rs2856718 polymorphism and HBV-related HCC (HCC versus CHB) risk in heterozygous model (AG versus AA).

The potential association of the HLA-DQ rs2856718 and HBV-related HCC was investigated in four articles; among which, three articles were in Chinese population and one in Saudi Arabian population. The meta-analysis of a possible association between the HLA-DQ rs2856718 and risk of HBV-related HCC is summarized in Table 2. The result of the controls and HBV-related HCC cases indicated a strong association of HLA-DQ rs2856718 with HBV-related HCC susceptibility. When the two populations were combined together, the overall OR for dominant model contrast (AG + GG versus AA: OR = 0.54, 95% CI = 0.47–0.62, *P*_Z_ < 0.001), recessive contrast (GG versus AG + AA: OR = 0.60, 95% CI = 0.51–0.70, *P*_Z_ < 0.001), homozygous contrast (GG versus AA: OR = 0.44, 95% CI: 0.37–0.53, *P*_Z_ < 0.001), heterozygous contrast (AG versus AA: OR = 0.59, 95% CI: 0.52–0.68, *P*_Z_ < 0.001), and allele contrast (G versus A: OR = 0.64, 95% CI: 0.58–0.70, *P*_Z_ < 0.001) was significantly associated with HBV-related HCC (Figure 2(a)). Additionally, this risk was more significant in the Chinese population, but there was no association between the HLA-DQ rs2856718 and HBV-related HCC susceptibility in Saudi Arabian population. When the controls were CHB patients, only GG versus AA and G versus A of HLA-DQ rs2856718 were significantly associated with the risk of HBV-related HCC. However, subgroup analysis by ethnicity showed that HLA-DQ rs2856718 AG genotype had a significantly increased risk of HBV-related HCC among Saudi Arabian population (AG versus AA: OR = 2.00, 95% CI: 1.12–3.58, *P*_Z_ = 0.019) (Figure 2(b)).

### 3.3. Meta-Analysis of the Association between HLA-DQ rs9275572 and HBV-Related HCC Risk

**Figure 3:**
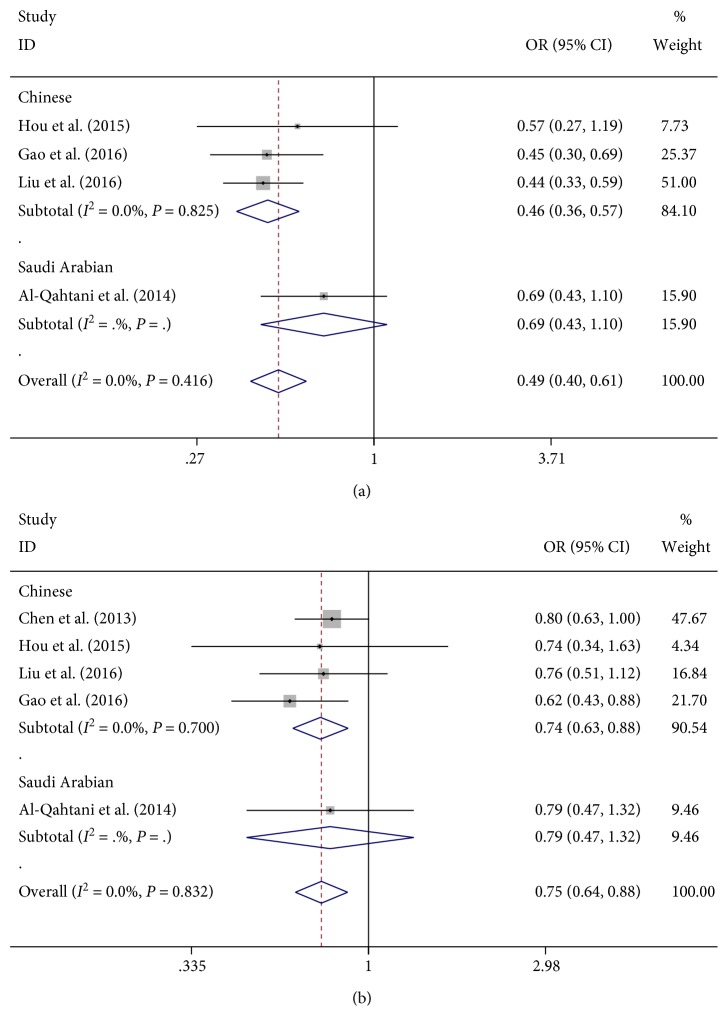
Forest plots for HLA-DQ rs9275572 polymorphism and the risk of HBV-related HCC. (a) Overall meta-analysis of the relationship between HLA-DQ rs9275572 polymorphism and HBV-related HCC (HCC versus control) risk in dominant model (AA + AG versus GG). (b) Overall meta-analysis of the relationship between HLA-DQ rs9275572 polymorphism and HBV-related HCC (HCC versus CHB) risk in dominant model (AA + AG versus GG).

Five studies reported a potential association between HLA-DQ rs9275572 polymorphism and HBV-related HCC risk with evidence from 1092 cases and 3566 controls (the healthy group and CHB group).  Table 3 shows the results for the association between HLA-DQ rs9275572 polymorphism and HBV-related HCC risk. When the controls were the healthy group, the HLA-DQ rs9275572 polymorphism was associated with decreased HBV-related HCC risk in all genetic models (AA + AG versus GG: OR = 0.49, 95% CI: 0.40–0.61, *P*_Z_ < 0.001; AA versus AG + GG: OR = 0.37, 95% CI: 0.24–0.56, *P*_Z_ < 0.001; AA versus GG: OR = 0.29, 95% CI: 0.19–0.45, *P*_Z_ < 0.001; AG versus GG: OR = 0.56, 95% CI: 0.45–0.69, *P*_Z_ < 0.001; and A versus G: OR = 0.52, 95% CI: 0.44–0.62, *P*_Z_ < 0.001), particularly in Chinese population, but not among Saudi Arabian population (Figure 3(a)). Similar results were found when the controls were the CHB group (AA + AG versus GG: OR = 0.75, 95% CI: 0.64–0.88, *P*_Z_ < 0.001; AA versus AG + GG: OR = 0.61, 95% CI: 0.42–0.87, *P*_Z_ = 0.007; AA versus GG: OR = 0.55, 95% CI: 0.38–0.80, *P*_Z_ = 0.002; AG versus GG: OR = 0.78, 95% CI: 0.66–0.93, *P*_Z_ = 0.005; and A versus G: OR = 0.76, 95% CI: 0.67–0.87, *P*_Z_ < 0.001) (Table 3 and Figure 3(b)).

### 3.4. Sensitivity Analysis

**Figure 4:**
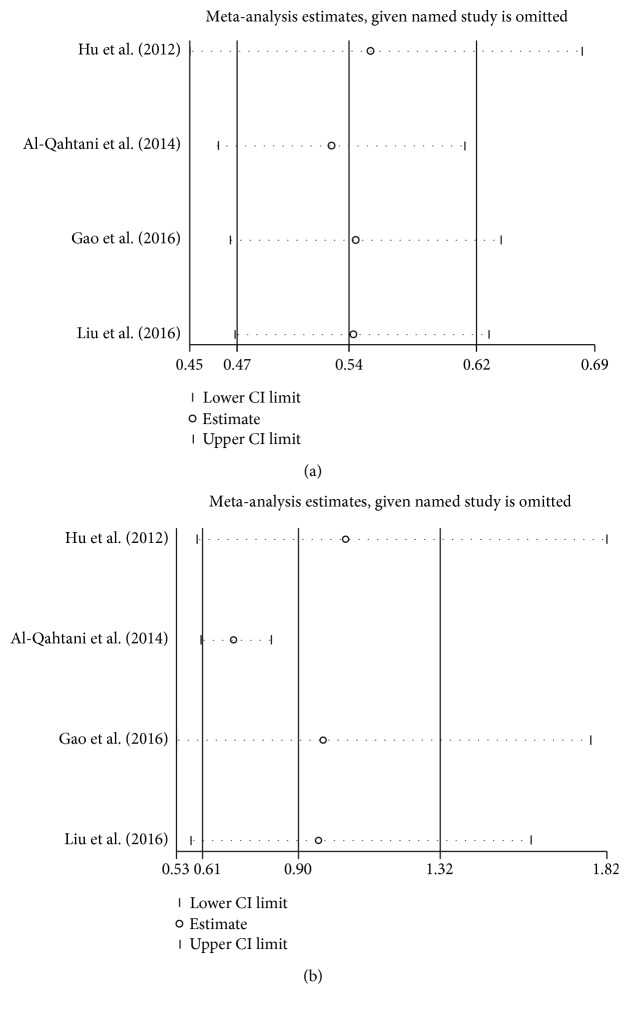
Sensitivity analysis of the pooled ORs and 95% CIs for HLA-DQ rs2856718 polymorphism. (a) The sensitivity analysis results of rs2856718 with HBV-related HCC (HCC versus control) in dominant model (AG + GG versus AA). (b) The sensitivity analysis results of rs2856718 with HBV-related HCC (HCC versus CHB) in heterozygous model (AG versus AA).

**Figure 5:**
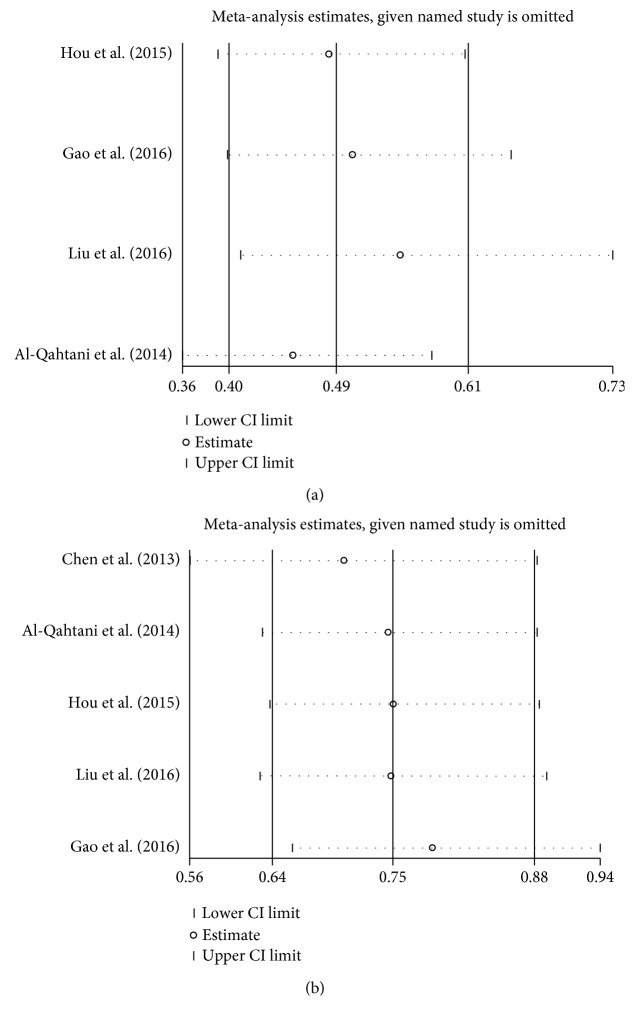
Sensitivity analysis of the pooled ORs and 95% CIs for HLA-DQ rs9275572 polymorphism. (a) The sensitivity analysis results of rs9275572 with HBV-related HCC (HCC versus control) in dominant model (AA + AG versus GG). (b) The sensitivity analysis results of rs9275572 with HBV-related HCC (HCC versus CHB) in dominant model (AA + AG versus GG).

The sensitivity analysis was performed to assess the influence of an individual study on the overall OR, and the corresponding pooled ORs were not materially altered (Figures 4 and 5).

### 3.5. Publication Bias

Publication bias of the included articles was assessed using Egger's test. The results of Egger's test indicated that no evidence of publication bias was observed in HLA-DQ (rs2856718 and rs9275572) polymorphisms (Tables 2 and 3).

## 4. Discussion

HLA-DQs belong to HLA class II molecules, which are expressed as cell-surface glycoproteins that present viral peptides to CD4^+^ T cells resulting in generating immunity against infection [20]. A study showed that the CD4^+^ T cells were significantly increased in tumor, ascites, and peripheral blood of patients with HCC and showed that HBV-specific and HLA class II-restricted CD4^+^ T cell responses may be related to HBV-related HCC development [21]. Recently, several studies have shown that genetic variations in HLA genes influence disease progression in HBV infection [22–25]. Accumulating evidence indicated the associations between HLA-DQ (rs2856718 and rs9275572) polymorphisms and HBV-related HCC, but the results are inconclusive or inconsistent. Hu et al.'s study showed that HLA-DQ rs2856718 polymorphism significantly decreased host HBV-related HCC risk in Southeast Han Chinese population [13]. Chen et al. confirmed that HLA-DQ rs9275572 polymorphism was significantly associated with HBV-related HCC risk in Chinese population [14], while Kumar et al. identified that it was associated with HCV-related HCC in Japanese patients [15]. Gao et al.'s study suggested that HLA-DQ (rs2856718 and rs9275572) polymorphisms were the risk factor of HBV-related HCC [18]. Other's study found that HLA-DQ rs2856718G and rs9275572A might be protective factors for HBV infection, HBV natural clearance, and HBV-related HCC progress [19]. However, Hou et al. found that HLA-DQ rs9275572 polymorphism was significantly different from the HBV-related HCC [17]. Meanwhile, a study observed that there was no association between HLA-DQ (rs2856718 and rs9275572) polymorphisms and HBV-related HCC in Saudi Arabian patients [16].

In order to resolve this conflict, we conducted a meta-analysis on the association between HLA-DQ (rs2856718 and rs9275572) polymorphisms and HBV-related HCC. Meta-analysis has been recognized as an important tool to more precisely define the effect of selected genetic polymorphisms on the risk for disease and to identify potentially important sources of between-study heterogeneity. There are variations between human populations, so a SNP allele that is common in one geographical or ethnic group may be much rarer in another. So we also analyzed the distribution of HLA-DQ (rs2856718 and rs9275572) polymorphisms in different ethnic groups. Our results indicated that HLA-DQ (rs2856718 and rs9275572) polymorphisms were associated with the decreased risk of HBV-related HCC in Chinese population, but not in Saudi Arabian population. However, the sample size in Saudi Arabian population is too small to conclude a negative association. The comparison between Chinese population and Saudi Arabian population may be imbalanced as a result of the sample size. Therefore, the findings of this study should be validated in the future through a population-based study.

As a meta-analysis of observational studies, there are some limitations. Firstly, we did not have original data for all studies to adjust estimates and perform a more precise analysis, including gender, age, drinking, smoking, lifestyle, body mass index, and so on. Secondly, the number of published studies was not sufficiently large for a comprehensive analysis. Thirdly, the interaction of gene-gene and of gene-environment has not been evaluated owing to the absence of original data. Therefore, more studies are needed to get more reliable results.

In conclusion, the current meta-analysis suggested that HLA-DQ (rs2856718 and rs9275572) polymorphisms were associated with HBV-related HCC risk among Chinese population. Taken together, our study suggested that HLA-DQ loci are candidate susceptibility regions that have some marker SNPs (rs2856718 and rs9275572) for HBV-related HCC in Chinese population.

## Figures and Tables

**Table 1:** Characteristics of the studies included in the meta-analysis.

First author	Year	Ethnicity	Genotyping method	Polymorphisms	Number of HCC	Number of CHB	Number of controls	HCC			CHB			Control			*P*_HWE_
								GG	AG	AA	GG	AG	AA	GG	AG	AA	
Hu [13]	2012	Chinese	TaqMan	rs2856718	1287	1334	1335	229	551	507	240	674	420	331	660	344	0.684
Chen [14]	2013	Chinese	TaqMan	rs9275572	506	772	—	319	170	17	445	277	50	—	—	—	0.438
Al-Qahtani [16]	2014	Saudi	DNA sequencing	rs2856718	71	603	505	18	31	22	184	173	246	155	223	127	0.010
		Arabian	TaqMan	rs9275572	81	208	571	39	34	8	88	95	25	223	251	97	0.069
Hou [17]	2015	Chinese	TaqMan	rs9275572	43	148	316	33	9	1	105	35	8	206	96	14	0.515
Gao [18]	2016	Chinese	Flight mass spectrometry	rs2856718	308	217	507	36	126	146	33	96	88	119	225	163	0.017
				rs9275572	308	396	505	212	84	12	245	129	22	249	196	60	0.029
Liu [19]	2016	Chinese	Flight mass spectrometry	rs2856718	154	112	254	19	62	73	17	50	45	61	112	81	0.073
				rs9275572	154	396	254	105	43	6	245	129	22	125	99	30	0.135

**Table 2:** Main results of the meta-analysis of the association between HLA-DQ (rs2856718) polymorphisms and the risk of HBV-related HCC.

Comparison	Subgroup	OR (95% CI)	Model	Heterogeneity test		*P*_Z_	*P*_E_
				*I* ^2^ (%)	*P*_H_		
HCC versus control							
Dominant model (AG + GG versus AA)	Overall	0.54 (0.47–0.62)	F	0	0.688	<0.001	0.41
	Chinese	0.53 (0.46–0.61)	F	0	0.990	<0.001	0.06
	Saudi Arabian	0.74 (0.44–1.29)	^∗^	^∗^	^∗^	0.294	^∗^
Recessive model (GG versus AG + AA)	Overall	0.60 (0.51–0.70)	F	42.7	0.155	<0.001	0.51
	Chinese	0.59 (0.50–0.69)	R	55.6	0.105	<0.001	0.22
	Saudi Arabian	0.77 (0.43–1.35)	^∗^	^∗^	^∗^	0.36	^∗^
Homozygous model (GG versus AA)	Overall	0.44 (0.37–0.53)	F	23.2	0.271	<0.001	0.82
	Chinese	0.43 (0.36–0.51)	F	13.7	0.314	<0.001	0.24
	Saudi Arabian	0.67 (0.34–1.30)	^∗^	^∗^	^∗^	0.24	^∗^
Heterozygous model (AG versus AA)	Overall	0.59 (0.52–0.68)	F	0	0.704	<0.001	0.09
	Chinese	0.58 (0.50–0.68)	F	0	0.841	<0.001	0.32
	Saudi Arabian	0.80 (0.45–1.45)	^∗^	^∗^	^∗^	0.46	^∗^
Allele model (G versus A)	Overall	0.64 (0.58–0.70)	F	23.0	0.273	<0.001	0.88
	Chinese	0.63 (0.57–0.69)	F	9.8	0.330	<0.001	0.22
	Saudi Arabian	0.80 (0.50–1.14)	^∗^	^∗^	^∗^	0.21	^∗^
HCC versus CHB							
Dominant model (AG + GG versus AA)	Overall	0.83 (0.63–1.10)	R	60.4%	0.056	0.194	0.308
	Chinese	0.72 (0.62–0.83)	F	0	0.093	<0.001	0.283
	Saudi Arabian	1. 53 (0.90–2.60)	^∗^	^∗^	^∗^	0.112	^∗^
Recessive model (GG versus AG + AA)	Overall	0.92 (0.78–1.09)	F	0	0.626	0.342	0.058
	Chinese	0.94 (0.78–1.12)	F	0	0.511	0.485	0.251
	Saudi Arabian	0.77 (0.44–1.36)	F	0	^∗^	0.370	^∗^
Homozygous model (GG versus AA)	Overall	0.79 (0.69–0.95)	F	0	0.676	0.014	0.982
	Chinese	0.78 (0.63–0.93)	F	0	0.796	0.008	0.271
	Saudi Arabian	1.09 (0.57–2.10)	F	0	^∗^	0.787	^∗^
Heterozygous model (AG versus AA)	Overall	0.90 (0.61–1.32)	R	75.9	0.006	0.579	0.245
	Chinese	0.70 (0.60–0.81)	F	0	0.719	<0.001	0.281
	Saudi Arabian	2.00 (1.12–3.58)	F	0	^∗^	0.019	^∗^
Allele model (G versus A)	Overall	0.85 (0.78–0.94)	F	0	0.486	0.001	0.707
	Chinese	0.84 (0.76–0.92)	F	0	0.887	<0.001	0.229
	Saudi Arabian	1.10 (0.77–1.56)	F	0	^∗^	0.599	^∗^

OR: odds ratio; CI: confidence interval; *P*_H_: *P* value of heterogeneity test; *P*_Z_: *P* value of Z test; *P*_E_: *P* value of Egger's test; R: random effect model; F: fixed effect model. ^∗^Because there was only one study with this genotype of rs2856718, the value could not be calculated.

**Table 3:** Main results of the meta-analysis of the association between HLA-DQ (rs9275572) polymorphisms and the risk of HBV-related HCC.

Comparison	Subgroup	OR (95% CI)	Model	Heterogeneity test		*P*_Z_	*P*_E_
				*I* ^2^ (%)	*P*_H_		
HCC versus control							
Dominant model (AA + AG versus GG)	Overall	0.49 (0.40–0.61)	F	0	0.416	<0.001	0.391
	Chinese	0.46 (0.36–0.57)	F	0	0.825	<0.001	0.168
	Saudi Arabian	0.69 (0.43–1.10)	^∗^	^∗^	^∗^	0.120	^∗^
Recessive model (AA versus AG + GG)	Overall	0.37 (0.24–0.56)	F	0	0.659	<0.001	0.698
	Chinese	0.31 (0.19–0.52)	F	0	0.885	<0.001	0.216
	Saudi Arabian	0.54 (0.25–1.15)	^∗^	^∗^	^∗^	0.108	^∗^
Homozygous model (AA versus GG)	Overall	0.29 (0.19–0.45)	F	0	0.537	<0.001	0.652
	Chinese	0.25 (0.15–0.41)	F	0	0.842	<0.001	0.208
	Saudi Arabian	0.47 (0.27–1.05)	^∗^	^∗^	^∗^	0.065	^∗^
Heterozygous model (AG versus GG)	Overall	0.56 (0.45–0.69)	F	0	0.525	<0.001	0.491
	Chinese	0.52 (0.40–0.66)	F	0	0.939	<0.001	0.097
	Saudi Arabian	0.77 (0.47–1.27)	^∗^	^∗^	^∗^	0.311	^∗^
Allele model (A versus G)	Overall	0.52 (0.44–0.62)	F	0	0.280	<0.001	0.501
	Chinese	0.48 (0.40–0.58)	F	0	0.782	<0.001	0.165
	Saudi Arabian	0.70 (0.49–1.00)	^∗^	21.8	^∗^	0.048	^∗^
HCC versus CHB							
Dominant model (AA + AG versus GG)	Overall	0.75 (0.64–0.88)	F	0	0.832	<0.001	0.674
	Chinese	0.74 (0.63–0.88)	F	0	0.700	0.001	0.616
	Saudi Arabian	0.79 (0.47–1.32)	^∗^	^∗^	^∗^	0.369	^∗^
Recessive model (AA versus AG + GG)	Overall	0.61 (0.42–0.87)	F	0	0.885	0.007	0.780
	Chinese	0.57 (0.38–0.85)	F	0	0.882	0.006	0.877
	Saudi Arabian	0.80 (0.35–1.86)	^∗^	^∗^	^∗^	0.608	^∗^
Homozygous model (AA versus GG)	Overall	0.55 (0.38–0.80)	F	0	0.934	0.002	0.766
	Chinese	0.52 (0.35–0.79)	F	0	0.934	0.002	0.897
	Saudi Arabian	0.72 (0.30–1.74)	^∗^	^∗^	^∗^	0.469	^∗^
Heterozygous model (AG versus GG)	Overall	0.78 (0.66–0.93)	F	0	0.727	0.005	0.651
	Chinese	0.78 (0.65–0.93)	F	0	0.566	0.007	0.649
	Saudi Arabian	0.81 (0.47–1.39)	^∗^	^∗^	^∗^	0.441	^∗^
Allele model (A versus G)	Overall	0.76 (0.67–0.87)	F	0	0.916	<0.001	0.750
	Chinese	0.75 (0.65–0.87)	F	0	0.869	<0.001	0.529
	Saudi Arabian	0.83 (0.57–1.23)	^∗^	^∗^	^∗^	0.362	^∗^

OR: odds ratio; CI: confidence interval, *P*_H_: *P* value of heterogeneity test; *P*_Z_: *P* value of Z test; *P*_E_: *P* value of Egger's test; R: random effect model; F: fixed effect model. ^∗^Because there was only one study with this genotype of rs9275572, the value could not be calculated.
